# (2*Z*)-*N*-(2-Chloro­benz­yl)-2-(2-oxo-2,3-dihydro-1*H*-indol-3-yl­idene)hydrazinecarbothio­amide

**DOI:** 10.1107/S1600536812035076

**Published:** 2012-08-23

**Authors:** Humayun Pervez, Nazia Khan, Mohammad S. Iqbal, Muhammad Yaqub, M. Nawaz Tahir

**Affiliations:** a Department of Chemistry, Bahauddin Zakariya University, Multan 60800, Pakistan; b Department of Chemistry, Forman Christian College, Lahore 54600, Pakistan; c University of Sargodha, Department of Physics, Sargodha, Pakistan

## Abstract

In the title compound, C_16_H_13_ClN_4_OS, the isatin ring system is oriented at dihedral angles of 10.60 (7) and 72.60 (3)° with respect to the thio­semicarbazide and 2-chloro­benzyl groups, respectively. The near planarity of the isatin and thio­semicarbazide groups [r.m.s. deviations of 0.0420 and 0.0163 Å, respectively] is reinforced by intra­molecular N—H⋯O and N—H⋯N hydrogen bonds, which generate *S*(6) and *S*(5) rings, respectively. In the crystal, inversion dimers linked by pairs of N—H⋯O hydrogen bonds generate *R*_2_^2^(8) loops. Aromatic π–π stacking inter­actions between the centroids of heterocyclic five-membered and benzene rings [distance = 3.6866 (11) Å] are also observed.

## Related literature

For biochemical background to isatins, see: Pervez *et al.* (2012 ▶). For a related structure, see: Ramzan *et al.* (2010 ▶). For graph-set notation, see: Bernstein *et al.* (1995 ▶).
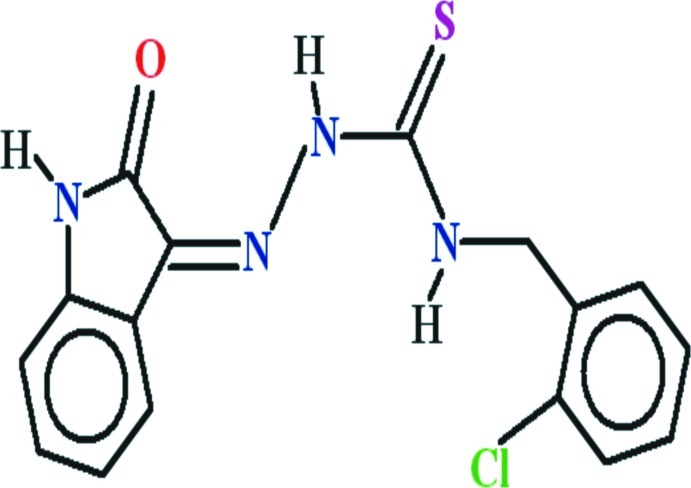


## Experimental

### 

#### Crystal data

C_16_H_13_ClN_4_OS
*M* *_r_* = 344.81
Monoclinic, 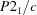

*a* = 13.7017 (10) Å
*b* = 14.1585 (10) Å
*c* = 8.2698 (5) Å
β = 93.151 (3)°
*V* = 1601.88 (19) Å^3^
*Z* = 4
Mo *K*α radiation
μ = 0.38 mm^−1^
*T* = 296 K
0.32 × 0.23 × 0.20 mm

#### Data collection

Bruker Kappa APEXII CCD diffractometer
Absorption correction: multi-scan (*SADABS*; Bruker, 2005 ▶) *T*_min_ = 0.888, *T*_max_ = 0.929
14957 measured reflections
3935 independent reflections
2541 reflections with *I* > 2σ(*I*)
*R*_int_ = 0.033

#### Refinement

*R*[*F* ^2^ > 2σ(*F* ^2^)] = 0.043
*wR*(*F* ^2^) = 0.105
*S* = 1.02
3935 reflections
208 parameters
H-atom parameters constrained
Δρ_max_ = 0.21 e Å^−3^
Δρ_min_ = −0.29 e Å^−3^

### 

Data collection: *APEX2* (Bruker, 2009 ▶); cell refinement: *SAINT* (Bruker, 2009 ▶); data reduction: *SAINT*; program(s) used to solve structure: *SHELXS97* (Sheldrick, 2008 ▶); program(s) used to refine structure: *SHELXL97* (Sheldrick, 2008 ▶); molecular graphics: *ORTEP-3 for Windows* (Farrugia, 1997 ▶) and *PLATON* (Spek, 2009 ▶); software used to prepare material for publication: *WinGX* (Farrugia, 1999 ▶) and *PLATON*.

## Supplementary Material

Crystal structure: contains datablock(s) global, I. DOI: 10.1107/S1600536812035076/hb6928sup1.cif

Structure factors: contains datablock(s) I. DOI: 10.1107/S1600536812035076/hb6928Isup2.hkl

Supplementary material file. DOI: 10.1107/S1600536812035076/hb6928Isup3.cml

Additional supplementary materials:  crystallographic information; 3D view; checkCIF report

## Figures and Tables

**Table 1:** Hydrogen-bond geometry (Å, °)

*D*—H⋯*A*	*D*—H	H⋯*A*	*D*⋯*A*	*D*—H⋯*A*
N3—H3*A*⋯O1	0.86	2.05	2.7416 (19)	137
N4—H4*A*⋯N2	0.86	2.28	2.663 (2)	107
N1—H1⋯O1^i^	0.86	2.09	2.903 (2)	157

Symmetry code: (i) 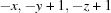.

**Fig. 1.**
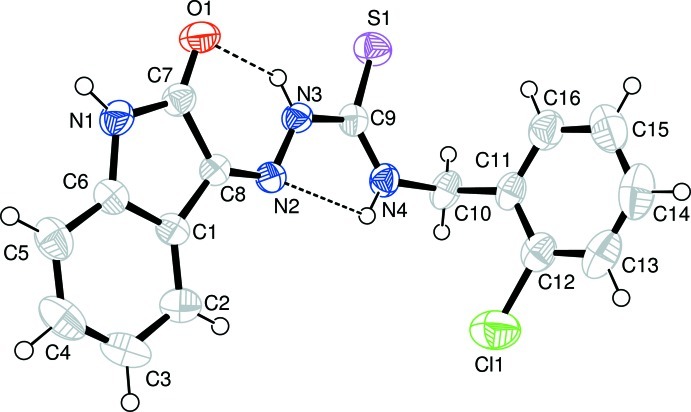
View of the title compound with displacement ellipsoids drawn at the 50% probability level. The dotted lines represent the intramolecular hydrogen bonds.

**Fig. 2.**
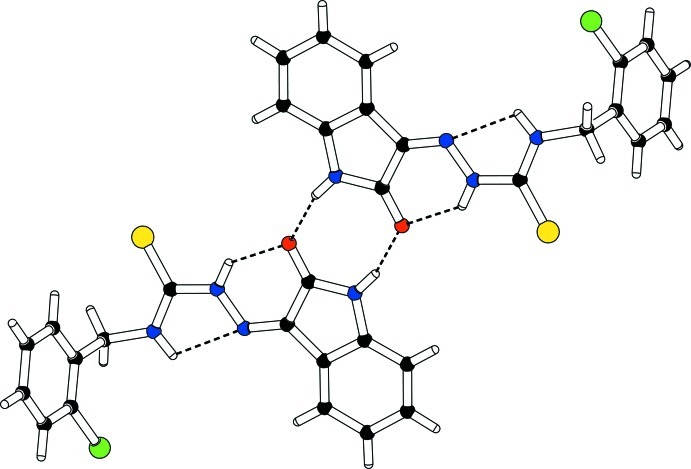
The partial packing diagram, which shows that molecules form dimers with R22(8) ring motifs.

## supplementary crystallographic information

### Comment

As a part of our ongoing work on the synthesis of isatin-thiosemicarbazones having certain pharmaceutical applications (Pervez *et al.*, 2012), we report herein the synthesis and crystal structure of the title compound (Fig. 1). The crystal structure of 1-(2-oxoindolin-3-ylidene)-4-[2-(trifluoromethoxy)phenyl]thiosemicarbazide (Ramzan *et al.* 2010) has been published which is related to the title compound.

In the title compound, the group A (C1—C8/N1/O1) of isatin moiety, group B (N2/N3/C9/S1/N4) of thiosemicarbazide and group C (C10—C16/CL1) of 2-chlorobenzyl are planar with r.m.s. deviations of 0.0230 Å, 0.0420 Å and 0.0163 Å, respectively. The dihedral angle between A/B, A/C and B/C are 10.60 (7)°, 72.60 (3)° and 72.89 (3)°, respectively. In the title compound, *S*(5) and *S*(6) ring motifs (Bernstein *et al.*, 1995) are formed due to intramolecular H-bondings of N—H···N and N—H···O types, respectively (Table 1, Fig. 1). The molecules are stabilized in the form of dimers due to H-bonding of N—H···O type (Table 1, Fig. 2) with *R*_2_^2^(8) ring motif. There exists π–π interaction between *Cg*1···*Cg*2^i^ [i = *x*, 1/2 - *y*, -1/2 + *z*] at a distance of 3.6866 (11) Å, where *Cg*1 and *Cg*2 are the centroids of heterocyclic five membered ring (C1/C6/N1/C7/C8) and the benzene ring (C1—C6), respectively. Similarly, π–π interaction exists between *Cg*2···*Cg*1^ii^ [ii = *x*, 1/2 - *y*, 1/2 + *z*] at a distance of 3.6866 (11) Å.

### Experimental

To a hot solution of isatin (0.74 g, 5 mmol) in 50% aqueous ethanol (15 ml) was added 4-(2-chlorobenzyl)thiosemicarbazide (1.08 g, 5 mmol) dissolved in ethanol (10 ml) under stirring. The reaction mixture was then refluxed for 2 h. The orange crystalline solid formed during heating was collected by suction filtration. Thorough washing with hot aqueous ethanol afforded the title compound in pure form (1.55 g, 90%), m.p. 525 K. The orange prisms of title compound were grown from chloroform solution by slow evaporation of the solvent.

### Refinement

The H-atoms were positioned geometrically at C—H = 0.93 Å and N—H = 0.86 Å, respectively and included in the refinement as riding with *U*_iso_(H) = *xU*_eq_(C, N), where *x* = 1.2 for all H-atoms.

### Crystal data

**Table:**

C_16_H_13_ClN_4_OS	*F*(000) = 712
*M**_r_* = 344.81	*D*_x_ = 1.430 Mg m^−^^3^
Monoclinic, *P*2_1_/*c*	Mo *K*α radiation, λ = 0.71073 Å
Hall symbol: -P 2ybc	Cell parameters from 2075 reflections
*a* = 13.7017 (10) Å	θ = 1.5–28.4°
*b* = 14.1585 (10) Å	µ = 0.38 mm^−^^1^
*c* = 8.2698 (5) Å	*T* = 296 K
β = 93.151 (3)°	Prism, orange
*V* = 1601.88 (19) Å^3^	0.32 × 0.23 × 0.20 mm
*Z* = 4	

### Data collection

**Table:**

Bruker Kappa APEXII CCD diffractometer	3935 independent reflections
Radiation source: fine-focus sealed tube	2541 reflections with *I* > 2σ(*I*)
Graphite monochromator	*R*_int_ = 0.033
Detector resolution: 7.50 pixels mm^-1^	θ_max_ = 28.4°, θ_min_ = 1.5°
ω scans	*h* = −18→18
Absorption correction: multi-scan (*SADABS*; Bruker, 2005)	*k* = −13→18
*T*_min_ = 0.888, *T*_max_ = 0.929	*l* = −8→11
14957 measured reflections	

### Refinement

**Table:**

Refinement on *F*^2^	Primary atom site location: structure-invariant direct methods
Least-squares matrix: full	Secondary atom site location: difference Fourier map
*R*[*F*^2^ > 2σ(*F*^2^)] = 0.043	Hydrogen site location: inferred from neighbouring sites
*wR*(*F*^2^) = 0.105	H-atom parameters constrained
*S* = 1.02	*w* = 1/[σ^2^(*F*_o_^2^) + (0.0415*P*)^2^ + 0.3273*P*] where *P* = (*F*_o_^2^ + 2*F*_c_^2^)/3
3935 reflections	(Δ/σ)_max_ < 0.001
208 parameters	Δρ_max_ = 0.21 e Å^−^^3^
0 restraints	Δρ_min_ = −0.29 e Å^−^^3^

### Special details

**Table:**

Geometry. Bond distances, angles *etc*. have been calculated using the rounded fractional coordinates. All su's are estimated from the variances of the (full) variance-covariance matrix. The cell e.s.d.'s are taken into account in the estimation of distances, angles and torsion angles
Refinement. Refinement of *F*^2^ against ALL reflections. The weighted *R*-factor *wR* and goodness of fit *S* are based on *F*^2^, conventional *R*-factors *R* are based on *F*, with *F* set to zero for negative *F*^2^. The threshold expression of *F*^2^ > σ(*F*^2^) is used only for calculating *R*-factors(gt) *etc*. and is not relevant to the choice of reflections for refinement. *R*-factors based on *F*^2^ are statistically about twice as large as those based on *F*, and *R*- factors based on ALL data will be even larger.

### Fractional atomic coordinates and isotropic or equivalent isotropic displacement parameters (Å^2^)

**Table:**

	*x*	*y*	*z*	*U*_iso_*/*U*_eq_	
Cl1	0.34601 (5)	−0.04349 (4)	0.12764 (8)	0.0798 (3)	
S1	0.30114 (4)	0.37338 (4)	−0.01319 (6)	0.0502 (2)	
O1	0.07991 (10)	0.44729 (9)	0.35390 (16)	0.0505 (5)	
N1	−0.05032 (11)	0.37716 (11)	0.47406 (17)	0.0434 (5)	
N2	0.11200 (10)	0.24682 (10)	0.24169 (15)	0.0336 (4)	
N3	0.17023 (10)	0.31195 (10)	0.17804 (16)	0.0363 (5)	
N4	0.26056 (11)	0.19523 (11)	0.06682 (17)	0.0402 (5)	
C1	−0.02814 (12)	0.22197 (12)	0.40963 (19)	0.0352 (5)	
C2	−0.04817 (15)	0.12656 (14)	0.4110 (2)	0.0488 (7)	
C3	−0.12841 (17)	0.09610 (16)	0.4929 (3)	0.0615 (8)	
C4	−0.18653 (16)	0.16013 (18)	0.5692 (3)	0.0628 (8)	
C5	−0.16717 (15)	0.25630 (16)	0.5693 (2)	0.0517 (7)	
C6	−0.08666 (13)	0.28509 (13)	0.48984 (19)	0.0382 (6)	
C7	0.02986 (13)	0.37795 (13)	0.3854 (2)	0.0380 (6)	
C8	0.04609 (12)	0.27811 (12)	0.33533 (18)	0.0329 (5)	
C9	0.24367 (12)	0.28702 (12)	0.07912 (19)	0.0351 (5)	
C10	0.34135 (14)	0.15508 (15)	−0.0198 (2)	0.0469 (7)	
C11	0.42635 (14)	0.12971 (14)	0.0949 (2)	0.0424 (6)	
C12	0.43573 (15)	0.04174 (14)	0.1681 (2)	0.0493 (7)	
C13	0.51332 (19)	0.01890 (19)	0.2740 (3)	0.0655 (9)	
C14	0.5822 (2)	0.0854 (2)	0.3117 (3)	0.0775 (10)	
C15	0.57512 (18)	0.1745 (2)	0.2452 (3)	0.0716 (10)	
C16	0.49822 (16)	0.19569 (16)	0.1363 (3)	0.0563 (8)	
H1	−0.07566	0.42658	0.51532	0.0520*	
H2	−0.00915	0.08379	0.35873	0.0585*	
H3	−0.14309	0.03203	0.49624	0.0737*	
H3A	0.16137	0.37063	0.19967	0.0436*	
H4	−0.24022	0.13810	0.62202	0.0754*	
H4A	0.22182	0.15693	0.11237	0.0482*	
H5	−0.20661	0.29916	0.62051	0.0620*	
H10A	0.36248	0.20052	−0.09843	0.0563*	
H10B	0.31882	0.09901	−0.07802	0.0563*	
H13	0.51841	−0.04121	0.31903	0.0786*	
H14	0.63470	0.07069	0.38322	0.0930*	
H15	0.62188	0.22004	0.27339	0.0861*	
H16	0.49455	0.25549	0.08983	0.0676*	

### Atomic displacement parameters (Å^2^)

**Table:**

	*U*^11^	*U*^22^	*U*^33^	*U*^12^	*U*^13^	*U*^23^
Cl1	0.0945 (5)	0.0626 (4)	0.0842 (4)	−0.0105 (3)	0.0230 (4)	0.0024 (3)
S1	0.0545 (3)	0.0484 (3)	0.0491 (3)	−0.0073 (3)	0.0144 (2)	0.0011 (2)
O1	0.0604 (9)	0.0332 (7)	0.0602 (8)	−0.0045 (7)	0.0230 (7)	−0.0027 (6)
N1	0.0451 (10)	0.0386 (9)	0.0477 (9)	0.0048 (7)	0.0148 (7)	−0.0051 (7)
N2	0.0338 (8)	0.0342 (8)	0.0326 (7)	0.0000 (7)	−0.0002 (6)	−0.0001 (6)
N3	0.0378 (9)	0.0321 (8)	0.0396 (8)	0.0006 (7)	0.0073 (7)	−0.0032 (6)
N4	0.0364 (9)	0.0423 (9)	0.0424 (8)	0.0042 (7)	0.0080 (7)	0.0018 (7)
C1	0.0363 (10)	0.0384 (10)	0.0304 (8)	−0.0040 (8)	−0.0014 (7)	−0.0002 (7)
C2	0.0574 (13)	0.0422 (11)	0.0466 (11)	−0.0061 (10)	0.0023 (9)	−0.0018 (9)
C3	0.0712 (16)	0.0534 (13)	0.0598 (13)	−0.0266 (12)	0.0038 (12)	0.0019 (11)
C4	0.0571 (14)	0.0799 (17)	0.0526 (12)	−0.0262 (13)	0.0135 (11)	0.0042 (12)
C5	0.0434 (12)	0.0663 (14)	0.0460 (11)	−0.0069 (11)	0.0091 (9)	−0.0059 (10)
C6	0.0381 (11)	0.0440 (10)	0.0323 (9)	−0.0036 (9)	0.0007 (8)	−0.0011 (8)
C7	0.0415 (11)	0.0363 (10)	0.0366 (9)	0.0018 (9)	0.0057 (8)	−0.0005 (7)
C8	0.0350 (10)	0.0340 (9)	0.0295 (8)	0.0011 (8)	−0.0010 (7)	−0.0008 (7)
C9	0.0343 (10)	0.0420 (10)	0.0287 (8)	0.0015 (8)	−0.0015 (7)	−0.0040 (7)
C10	0.0504 (12)	0.0531 (12)	0.0376 (10)	0.0165 (10)	0.0065 (9)	−0.0019 (9)
C11	0.0421 (12)	0.0499 (11)	0.0364 (9)	0.0132 (10)	0.0121 (8)	−0.0004 (8)
C12	0.0519 (13)	0.0535 (12)	0.0439 (11)	0.0106 (10)	0.0159 (10)	−0.0010 (9)
C13	0.0745 (17)	0.0705 (16)	0.0519 (13)	0.0304 (15)	0.0065 (12)	0.0110 (11)
C14	0.0614 (17)	0.103 (2)	0.0668 (16)	0.0278 (17)	−0.0094 (13)	−0.0021 (15)
C15	0.0481 (14)	0.090 (2)	0.0766 (16)	0.0000 (14)	0.0015 (12)	−0.0178 (15)
C16	0.0509 (14)	0.0575 (14)	0.0615 (13)	0.0094 (12)	0.0111 (11)	0.0021 (10)

### Geometric parameters (Å, º)

**Table:**

Cl1—C12	1.742 (2)	C5—C6	1.376 (3)
S1—C9	1.6625 (18)	C7—C8	1.493 (2)
O1—C7	1.234 (2)	C10—C11	1.505 (3)
N1—C6	1.404 (2)	C11—C16	1.387 (3)
N1—C7	1.354 (2)	C11—C12	1.388 (3)
N2—N3	1.345 (2)	C12—C13	1.378 (3)
N2—C8	1.299 (2)	C13—C14	1.357 (4)
N3—C9	1.377 (2)	C14—C15	1.378 (4)
N4—C9	1.325 (2)	C15—C16	1.381 (3)
N4—C10	1.466 (2)	C2—H2	0.9300
N1—H1	0.8600	C3—H3	0.9300
N3—H3A	0.8600	C4—H4	0.9300
N4—H4A	0.8600	C5—H5	0.9300
C1—C8	1.453 (2)	C10—H10A	0.9700
C1—C2	1.379 (3)	C10—H10B	0.9700
C1—C6	1.393 (2)	C13—H13	0.9300
C2—C3	1.391 (3)	C14—H14	0.9300
C3—C4	1.382 (3)	C15—H15	0.9300
C4—C5	1.387 (3)	C16—H16	0.9300
			
C6—N1—C7	111.13 (15)	C10—C11—C16	120.63 (18)
N3—N2—C8	116.49 (14)	C10—C11—C12	122.63 (18)
N2—N3—C9	121.67 (14)	C11—C12—C13	122.5 (2)
C9—N4—C10	123.90 (15)	Cl1—C12—C11	119.38 (15)
C7—N1—H1	124.00	Cl1—C12—C13	118.11 (17)
C6—N1—H1	124.00	C12—C13—C14	119.1 (2)
C9—N3—H3A	119.00	C13—C14—C15	120.7 (2)
N2—N3—H3A	119.00	C14—C15—C16	119.6 (2)
C9—N4—H4A	118.00	C11—C16—C15	121.4 (2)
C10—N4—H4A	118.00	C1—C2—H2	121.00
C2—C1—C6	120.31 (16)	C3—C2—H2	121.00
C2—C1—C8	133.12 (16)	C2—C3—H3	120.00
C6—C1—C8	106.56 (15)	C4—C3—H3	120.00
C1—C2—C3	118.08 (18)	C3—C4—H4	119.00
C2—C3—C4	120.6 (2)	C5—C4—H4	119.00
C3—C4—C5	122.0 (2)	C4—C5—H5	122.00
C4—C5—C6	116.66 (19)	C6—C5—H5	122.00
N1—C6—C1	109.58 (15)	N4—C10—H10A	109.00
N1—C6—C5	128.09 (17)	N4—C10—H10B	109.00
C1—C6—C5	122.33 (18)	C11—C10—H10A	109.00
O1—C7—N1	126.73 (17)	C11—C10—H10B	109.00
O1—C7—C8	127.01 (16)	H10A—C10—H10B	108.00
N1—C7—C8	106.26 (15)	C12—C13—H13	120.00
N2—C8—C7	127.38 (15)	C14—C13—H13	120.00
N2—C8—C1	126.22 (16)	C13—C14—H14	120.00
C1—C8—C7	106.39 (14)	C15—C14—H14	120.00
S1—C9—N3	117.66 (13)	C14—C15—H15	120.00
S1—C9—N4	126.66 (13)	C16—C15—H15	120.00
N3—C9—N4	115.68 (15)	C11—C16—H16	119.00
N4—C10—C11	111.33 (14)	C15—C16—H16	119.00
C12—C11—C16	116.71 (18)		
			
C7—N1—C6—C1	0.42 (19)	C1—C2—C3—C4	0.6 (3)
C7—N1—C6—C5	−179.26 (17)	C2—C3—C4—C5	−0.8 (4)
C6—N1—C7—O1	−177.87 (17)	C3—C4—C5—C6	−0.2 (3)
C6—N1—C7—C8	1.46 (18)	C4—C5—C6—N1	−178.93 (18)
C8—N2—N3—C9	178.97 (14)	C4—C5—C6—C1	1.4 (3)
N3—N2—C8—C1	176.21 (14)	O1—C7—C8—N2	−4.5 (3)
N3—N2—C8—C7	−2.4 (2)	O1—C7—C8—C1	176.61 (17)
N2—N3—C9—S1	171.76 (11)	N1—C7—C8—N2	176.14 (16)
N2—N3—C9—N4	−7.8 (2)	N1—C7—C8—C1	−2.73 (18)
C10—N4—C9—S1	6.5 (2)	N4—C10—C11—C12	89.5 (2)
C10—N4—C9—N3	−174.00 (14)	N4—C10—C11—C16	−88.4 (2)
C9—N4—C10—C11	98.6 (2)	C10—C11—C12—Cl1	−0.3 (2)
C6—C1—C2—C3	0.6 (3)	C10—C11—C12—C13	−179.54 (19)
C8—C1—C2—C3	−178.34 (19)	C16—C11—C12—Cl1	177.75 (15)
C2—C1—C6—N1	178.63 (15)	C16—C11—C12—C13	−1.5 (3)
C2—C1—C6—C5	−1.7 (3)	C10—C11—C16—C15	178.0 (2)
C8—C1—C6—N1	−2.16 (18)	C12—C11—C16—C15	−0.1 (3)
C8—C1—C6—C5	177.54 (16)	Cl1—C12—C13—C14	−177.65 (19)
C2—C1—C8—N2	3.1 (3)	C11—C12—C13—C14	1.7 (3)
C2—C1—C8—C7	−177.98 (18)	C12—C13—C14—C15	−0.1 (4)
C6—C1—C8—N2	−175.93 (15)	C13—C14—C15—C16	−1.4 (4)
C6—C1—C8—C7	2.96 (17)	C14—C15—C16—C11	1.5 (4)

### Hydrogen-bond geometry (Å, º)

**Table:**

*D*—H···*A*	*D*—H	H···*A*	*D*···*A*	*D*—H···*A*
N3—H3*A*···O1	0.86	2.05	2.7416 (19)	137
N4—H4*A*···N2	0.86	2.28	2.663 (2)	107
N1—H1···O1^i^	0.86	2.09	2.903 (2)	157

Symmetry code: (i) −*x*, −*y*+1, −*z*+1.
